# A nature inspired modularity function for unsupervised learning involving spatially embedded networks

**DOI:** 10.1038/s41598-019-39180-8

**Published:** 2019-02-22

**Authors:** Raj Kishore, Ajay K. Gogineni, Zohar Nussinov, Kisor K. Sahu

**Affiliations:** 1 School of Minerals, Metallurgical and Materials Engineering, Indian Institute of Technology Bhubaneswar-, Bhubaneswar, 752050 India; 2 School of Electrical Sciences, Indian Institute of Technology Bhubaneswar, Bhubaneswar, 752050 India; 3 Department of Physics, Washington University in Saint Louis, Saint Louis, MO 63130-4899 USA

## Abstract

The quality of network clustering is often measured in terms of a commonly used metric known as “modularity”. Modularity compares the clusters found in a network to those present in a random graph (a “null model”). Unfortunately, modularity is somewhat ill suited for studying spatially embedded networks, since a random graph contains no basic geometrical notions. Regardless of their distance, the null model assigns a nonzero probability for an edge to appear between any pair of nodes. Here, we propose a variant of modularity that does not rely on the use of a null model. To demonstrate the essentials of our method, we analyze networks generated from granular ensemble. We show that our method performs better than the most commonly used Newman-Girvan (NG) modularity in detecting the best (physically transparent) partitions in those systems. Our measure further properly detects hierarchical structures, whenever these are present.

## Introduction

One of the most successful strategies in understanding a complex scientific/engineering problem involves two essentially crucial steps: deconstruction and reconstruction. In the “deconstruction” stage, one breaks a complex, nearly intractable problem into many elementary parts. These basic constitutes may be then more readily studied. During reconstruction, one glues these smaller parts back together, as they appear in the original system. In reconstruction of the original system from its basic building blocks, one places emphasis on attempting to understand the response of the entire system as a whole. In the past, most deconstruction steps used to be performed manually (a step often somewhat facilitated by taking advantage of inherent symmetries of the problem whenever these were present). With the advancement of technology, the complexity and size of routinely investigated systems are constantly increasing. Consequently abstract and scientifically superior mathematical methods, like network partitioning are becoming increasingly popular. These methods play a more prominent role in the deconstruction step. Networks appear naturally and may efficiently represent numerous systems in diverse fields including the social sciences, biology, information technology, neurosciences and economics. The architectures of real physical spatially embedded networks where the nodes are defined by their positions in Euclidean space are highly influenced by geometric constraints. Such physical networks are fundamentally different from networks that are not anchored in Euclidean space^1–3^.

In the current work, we will examine a particular class of spatially embedded networks, which are obtained from granular materials. In this type of networks, both long distance edges and hubs (high degree nodes) are absent. Also, the total number of edges in such graphs scales linearly with the number of nodes. The absence of hubs renders the topologies relatively feature-less. This more monotonous structure poses a great challenge for general purpose partitioning algorithms. Network analysis of granular materials and their ensembles often facilitates a scientific description of the complex structure of interacting particles, responsible for many bulk properties like mechanical stability, acoustic and heat transmission^4–7^ and paves the path for automated data analytics tools. The first step in this approach is to represent a granular assembly as a network. Herein, each particle is depicted by a node. An edge between any two particles captures their physical contacts or interactions (e.g. force). The second step is to detect “community structures” in these networks by optimizing an objective function^8,9^ to extract any underlying mesoscale architecture.

The quality of partitioning a network, also known as clustering, is reflected in its ability to make a distinction between densely connected nodes of “communities” or “modules” that are sparsely linked to other communities. The most prevalent objective function used for this optimization is the so-called “modularity” of a network. A maximization of the modularity function leads to putative partitions of the network into clusters. Amongst many different modularity functions^10–13^, Newman-Girvan (NG) modularity^14,15^ is most commonly used for real networks^16–19^. The NG modularity contrasts the number of actual connections in a given network to that of a null model. The null model is obtained by randomly changing the network connections without altering degree of respective nodes. Conceptually, the construction of the null model can be achieved in the following way: (i) all the edges are cut in two and consequently (ii) any two half-edges are re-connected with equal probability. For single layer network, the NG modularity is given as

1 $${Q}_{NG}=\frac{1}{2m}\sum _{i,j}({A}_{ij}-\frac{{k}_{i}{k}_{j}}{2m})\delta ({\sigma }_{i},{\sigma }_{j})$$

Here, *m* is total number of edges in the network, *σ*_*i*_ denotes the community that node *i* belongs to, the numbers *k*_*i*_ and *k*_*j*_ are the degree of *i*^*th*^ and *j*^*th*^ node respectively (i.e the number of edges that respectively, have node *i* and *j* as an endpoint), *A*_*ij*_ is the adjacency matrix (*A*_*ij*_ = 1, if an edge between *i*^th^ and *j*^th^ node exist, and *A*_*ij*_ = 0 otherwise) and δ(*σ*_*i*_, *σ*_*i*_) is the Kronecker delta whose value will be unity if *i*^*th*^ and *j*^*th*^ node have the same community membership and zero otherwise.

Since the NG modularity contrasts the real connections in the given network with the randomized connection of the null model as stated above, it is well suited for the networks where connections between any two arbitrary nodes are, at least in principle, not forbidden. However in many physical systems in which the networks are embedded in finite three- or two- dimensional Euclidian space, the edges are distance dependent. In particular, in many such systems, edges between arbitrary distant sites do not appear (since there are essentially neither forces nor correlations between far separated particles or sites). Thus it seems somewhat inappropriate to penalize (i.e render the value of modularity lower) for the absence of edges between two nodes, which are not in close physical proximity. An edge between distant nodes is not physically possible (even though they might belong to same community). Therefore the null model should be restricted from making those connections for spatially embedded networks. Attempts have been made to adapt the modularity for spatially embedded static and temporal networks, though, invariably, some of the most important works^3,20–23^ uses null models. This is appropriate when, at least some knowledge exist for the systems under investigation.

With the rapid growth of modern technology, sensors and devices collect vast raw data. This engenders “the big data” problem in many different arenas. Often the volume of raw unlabeled data far outpaces the increase in labeled data, as in e.g, medical diagnostics. In many cases, even basic information about the field of study might not be available (or is economically unfavorable to harness for the purpose of supervised learning). It is very likely that unsupervised learning is going to play an increasingly vital role in analyzing “big data”. The quintessential example of unlabeled data systems that we study in this article describes granular materials. To the best of our knowledge, no labeled data of meaningful quantity exists in the public domain for these systems. This lack of labeled data occurs despite the fact that granular materials lie at the core of many industries including pharmaceutical, mining, mineral, chemical, fertilizer and agriculture^24–26^. Moreover, from the principle of objectivity, one can expect that sufficient information for optimal/nearly-optimal partitioning of a network can be sourced intrinsically.

Based on these considerations, here we propose a new physically inspired function, that is extremely simple and incorporates provisions for geometrical constraints for spatially embedded networks and demonstrate its suitability for use in unsupervised learning methods using granular network as a test case. Our model enables the detection and characterization of structures that vary over a broad range of length scales. As such, our model might be useful for many real world applications in which multiple length scales are significant.

## Methods

### Development of granular network

Granular networks for the present study have been created using two different protocols. The first protocol uses a physics-based Discrete Element Method (DEM) that simulates true granular dynamics. In the second protocol, we synthetically generate granular ensemble where, particles are positioned in a hexagonal lattice. We then use a square stencil to remove particles and therefore produce a patterned disordered region.

### DEM simulation to generate granular ensemble

**Figure 1:**
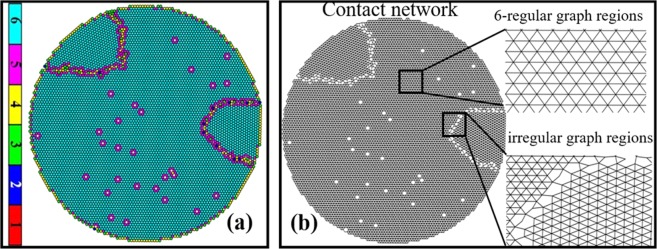
Granular ensemble and corresponding granular network. (**a**) Packing of 7428 uniform sized disks obtained by centripetal packing through DEM simulation (see main text and SI-1). The particles are colored based on their coordination numbers (see the colorbar at the extreme left). The ordered and disordered regions can be visually discerned. A good modularity function should become maximal for the partition that matches these partitions obtained by visual inspection. **(b)** Contact network formed from ensemble (**a**) by following the recipe: an edge is drawn between two particles if they are in physical contact. The weight of all edges is fixed as unity to make it an un-weighted network. Six-regular graph regions and irregular regions are shown in inset (extreme right). [The files containing node connectivity (graph_srep.gml) and the coordinates of each node and their corresponding degree (Particle_pos_with_coord_no_Srep.xls) can be downloaded from https://sites.google.com/a/iitbbs.ac.in/kks-research-work/research-data].

The granular ensemble of the present study is created using DEM^27–29^ simulation of 7428 macroscopic 2D particles of uniform size (radius 0.01 *m*) by centripetal packing. In this approach, an externally applied centripetal force (magnitude effectively equal to gravitational force), is directed towards the center of the box. DEM simulation protocol adopted for this article is identical to refs^30,31^, but nevertheless described in Supplementary Information (SI)-1 for the sake of completeness. All the particles move towards the center of the box because of this centripetal force and form a packing. During this packing process, collisions between particles cause reduction in their kinetic energy. Most of the particles in final structure pack in six-fold coordinated hexagonal structure. A very small fraction of the particles have coordination number (number of physically touching neighbors) different from six (Fig. 1a). The network is constructed by placing an edge between any two granules if they are in physical contact. Since most of the particles in the ensemble display six-fold coordination, the resultant network is a composition of mostly 6-regular sub-graph regions separated by irregular regions (Fig. 1b). The irregular regions consist of weakly connected nodes. These regions maybe visually discriminated from the strongly connected regular regions.

### Synthetically generated granular ensemble

In this protocol, particles are positioned in a regular hexagonal lattice. We then use a square stencil to remove particles and therefore produce a patterened disordered region. These patterns form logical boundaries for community partitions. The network formation protocol from these ensembles is same as discussed earlier.

### Finding the communities

For finding and creating communities in the network, we employ two different protocols. In the first protocol, we use a Potts model based community detection scheme discussed next. In the second protocol, we manually set the communities.

### Potts model based community detection algorithm

The accuracy of a graph clustering algorithm is mainly controlled by the quality function it uses. It should favor more edges inside community as well as restricts large number of missing edges by using some penalization function. We have used spin-glass-type Potts model algorithm for the community detection of Rhonhovde and Nussinov^9^ (hereafter referred as RN model) This algorithm is described in details in SI-2 and the complete c^++^ code of RN method can be downloaded from https://sites.google.com/a/iitbbs.ac.in/kks-research-work/research-data). The RN method attempts to iteratively find a partition that corresponds to the ground state of the following energy function (or Hamiltonian *H*) given in Eq. (2).

2 $$H(\{\sigma \})=-\,\frac{1}{2}\sum _{i\ne j}({a}_{ij}{A}_{ij}-\gamma {b}_{ij}{J}_{ij})\delta ({\sigma }_{i},{\sigma }_{j})$$

The asymmetry between connected (*A*_*ij*_ = 1) and disconnected (*J*_*ij*_ = *1-A*_*ij*_ = 1) edges may be reflected by individually setting the respective edge weights (denoted by *a* and *b*). The multiplicative factor, γ, is used as a structural resolution parameter. Altering the value of γ may increase or decrease the missing edge weights thus providing control over size and number of communities found by minimizing the Hamiltonian of Eq. (2). Typically, larger values of *γ* favor smaller communities, and vice versa.

The Potts Hamiltonian favors an intra-community link whereas it disfavors the missing edges within same community. The optimised state of the potts model Hamiltonian have mostly similar spin entities in same state and vice versa^32^.

### Manually setting the communities

In this protocol, we manually set the community boundaries without any regard to its optimality. If the community boundaries coincide with those associated with naturally occurring identifiable structures, clearly representing an optimal solution, then one may expect a higher modularity value, provided the function is properly constructed. At other times, we forcefully set the community boundaries not to coincide with such structures. Then one may anticipate a very low modularity values for such sub-optimal partitions. We will clearly demonstrate that our new modularity function is more sensitive to such changes and outperforms the NG modularity.

### New Modularity function

Before presenting our new function, we briefly discuss physical intuition that underlies our formulation. This intuition is deeply rooted in the study of naturally occurring magnetic materials. Specifically, the modularity function that we will shortly introduce is inspired by the formation of magnetic domains in ferromagnetic materials. In a magnetic material, there are “domains” in which all the moments tend to locally align (or become “polarized”) along the same direction. Such structures are of low energy. We wish to draw an analogy between such magnetic domains and clusters in a graph. Each node in the graph may be viewed as a local magnetic moments or “spin”, (such as that associated with an individual atom). An edge in the graph represents a magnetic interaction between two local moments (atoms/nodes). Along these lines, each community in the graph can indeed be viewed as a magnetic domain. Our modularity function is modeled by a Potts Hamiltonian^33^. The construction of this Hamiltonian essentially hinges upon the choices one makes to model the spin-spin interactions for four different scenarios. Adding these interactions together will yield the full Hamiltonian describing our system. The four possible scenarios in this spin dynamic formulation are the following (see SI-3 for details): (i) interaction between spins of identical polarization within same community (ii) interaction associated with a missing edge between nodes within the same community (i.e two nodes of identical spin polarization), (iii) interaction for an edge between two different communities (or two different magnetic domains of different spin polarization) and (iv) interaction representing a missing edge between two nodes from two separate communities. We will use a Heaviside step function to incorporate geometrical constraint associated with the individual items. A complete pictorial guide for all possible scenarios can be found in SI-4. While, the energy of a system (the Hamiltonian) is an extensive quantity, we will make the modularity an intensive parameter by scaling it with the total number of edges. We do so since, if the architecture of the network is relatively homogenous, we expect a comparable value of the modularity capturing the system size independent “quality” of the partition. Our proposed modularity function reads:

3 $$Q(\sigma )=\frac{1}{2m}\sum _{i,j}({a}_{ij}{A}_{ij}-\theta ({\rm{\Delta }}{x}_{ij})|{b}_{ij}|{J}_{ij})(2\delta ({\sigma }_{i},{\sigma }_{j})-1)$$

Here *a*_*ij*_ and *b*_*ij*_ are the strength (not to be confused with edge weights) of connected and missing edges between *i*^*th*^ and *j*^*th*^ nodes respectively. For setting up the strengths of edges, *a*_*ij*_ and *b*_*ij*_, there can be many choices. We employ a comparison to the local degree distribution between the *i*^*th*^ and *j*^*th*^ node with the average degree distribution 〈*k*〉 of the network, and set

4 $${a}_{ij}={b}_{ij}=(\frac{{k}_{i}+{k}_{j}}{2}-\langle k\rangle )$$

where, $\langle k\rangle =\frac{1}{N}\sum _{r=1}^{N}{k}_{r}$ and *N* is total number of nodes and *k* is the node degree.

5 $$\{2\delta ({\sigma }_{i},\,{\sigma }_{j})-1\}=\{\begin{array}{ll}\,\,1 & {\sigma }_{i}={\sigma }_{j}(i.e\,{\rm{in}}\,{\rm{same}}\,{\rm{community}})\\ -\,1 & {\sigma }_{i}\ne {\sigma }_{j}(i.e.\,{\rm{in}}\,{\rm{different}}\,\mathrm{communities})\end{array}\}$$

Our proposition, therefore, has two important distinctions from almost all of the traditional modularity functions that we know of, including the NG modularity (Eq. (1)) as well as other modularity functions developed for spatial networks. First, it does not explicitly resort to a null model (although the function of Eqs (2 and 3) was largely inspired by such a comparison). Second and most importantly, traditional methods neglect the inter-community interactions (both the existing and missing edges) whereas our function accounts for them. The key difference is embodied by the geometrical dependence that appears in our modularity of Eq. (3). The NG modularity penalizes for all missing edges between two distant nodes within same community. This penalty is imposed even when that edge might not be physically possible because of geometrical constraint. In order to restrict this incorrect over penalization, we introduced, in Eq. (3), a Heaviside unit step function *θ(Δx*_*ij*_) which incorporates the geometrical constraints in the form of neighborhood definition. Here ${\rm{\Delta }}{x}_{ij}={x}_{c}-|{\overrightarrow{r}}_{i}-{\overrightarrow{r}}_{j}|$ is the difference in Euclidian distance between nodes *i, j* and *x*_*c*_ defines cutoff distance for neighborhood and was chosen to be *x*_*c*_ = *1.05* * (*R*_*i*_ + *R*_*j*_) where, *R* is the radius of particle for the present study. The function *θ(Δx*_*ij*_) introduces a penalty only for those missing links where an edge is geometrically possible.

6 $$\theta ({\rm{\Delta }}{x}_{ij})=\{\begin{array}{ll}1 & {\rm{\Delta }}{x}_{ij} > 0\,\,(\mathrm{within}\,{\rm{cut}}\,\mathrm{off})\\ 0 & {\rm{otherwise}}\,(\mathrm{outside}\,{\rm{cut}}\,\mathrm{off})\end{array}\}$$

Our function compares the local degree distribution at node level with the average degree distribution of the network (Eq. (4)). Its value will be high if the nodes of a community are highly linked with each other. Highly linked communities exhibit a local degree distribution that is larger than the average degree distribution over the entire network. We remark that employing the absolute value |*b|*, in Eq. (3) is not mandatory; this takes care of a subtlety as we explained in SI-4. A thorough discussion on the implications of Eq. (3) for all possible scenarios is also exhaustively discussed therein.

## Results

Table 1 outlines our protocol combinations (PCs) and the associated key results. In this study, three types of two-dimensional (2-D) granular networks were prepared using different protocol combinations and their corresponding results are discussed in details. In PC-1 we performed realistic simulation of 2-D granular ensemble (using DEM method) that produced a natural 2-D ensemble (Fig. 1a) from which the network was generated (Fig. 1b). Communities in this network were found using RN method at different resolution values (γ). Modularity for each partition was calculated by both NG- and new- modularity functions. We first illustrate the role of this resolution parameter (γ) and further evaluate the performance of both NG and new modularity functions for finding the best partition. In PC-2, we analyzed a synthetically generated granular ensemble (i.e., without using DEM) with periodic distribution of disordered regions. RN method was used at different resolutions to find the partitions. Both the NG- and new- modularity functions were quizzed about the best partition. The results were compared to “rational human expectations”. In essence, we evaluate, which among these two functions more closely mimic “human-like” vision processing. In PC-3, we again used a synthetically generated granular ensemble. In this case, prime objective is to find which method is more capable in identifying the hierarchical organization. Therefore, the community boundaries were sometime set to coincide with natural boundaries and sometime not. This was done at different levels of hierarchical organizations, i.e., community size was varied from low to high (fine to coarse size).

**Table 1:** Usage of different combinations of protocols and organization of their results.

Protocol Combinations (PC)	Key findings	Protocols used for			Objectives
		Ensemble generation	Finding community	Modularity	
PC 1	Best partition	DEM	RN model. γ used.	NG vs. new	Discuss the role of γ in finding the optimal partition. Evaluate NG vs. new modularity for finding the best partition.
PC 2	Best partition	Synthetic (manual)	RN model. γ used.	NG vs. new	Evaluate NG vs. new modularity for finding the best partition.
PC 3	Hierarchical structures	Synthetic (manual)	Manual (forced). γ not used.	NG vs. new	Evaluate NG vs. new modularity for finding hierarchical structures.

### Results for PC 1

**Figure 2:**
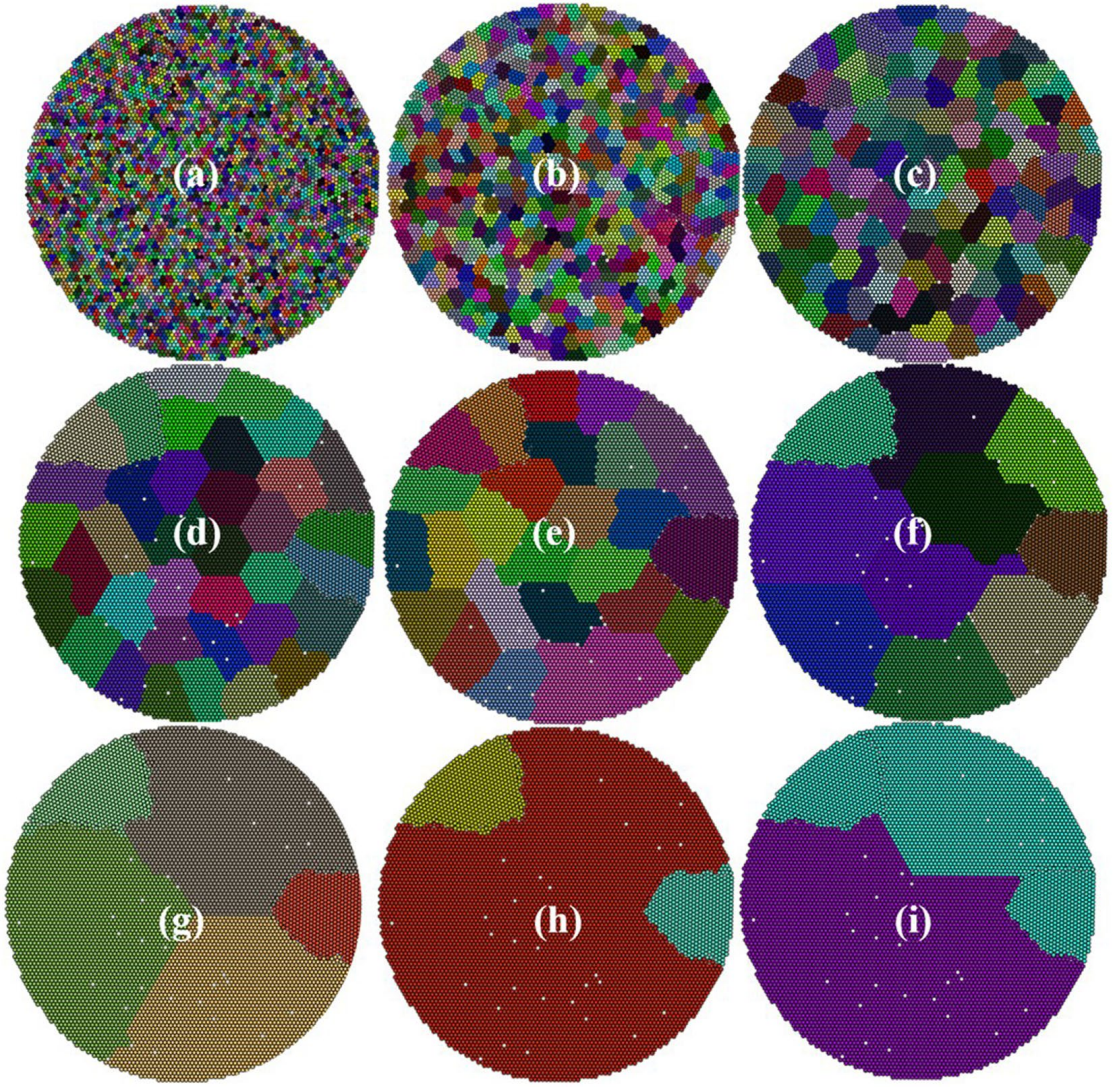
The community structures obtained at different *γ* values **(a**) 10 **(b)** 3 × 10^−1^ **(c)** 5 × 10^−2^ **(d)** 7 × 10^−3^ **(e)** 5 × 10^−3^ **(f)** 9 × 10^−4^ **(g)** 6 × 10^−4^ **(h)** 4 × 10^−4^ **(i)** 5 × 10^−5^. The edges are not shown for clarity. All the nodes of a community have been assigned same color to make them visually separable and the color for each community is randomly selected. By a visual comparison with Fig. 1a,b, one can immediately point that (**h**) shows the most optimal partition. In next figure, both NG- and new-modularity functions will be quizzed about the quality of these partitions.

**Figure 3:**
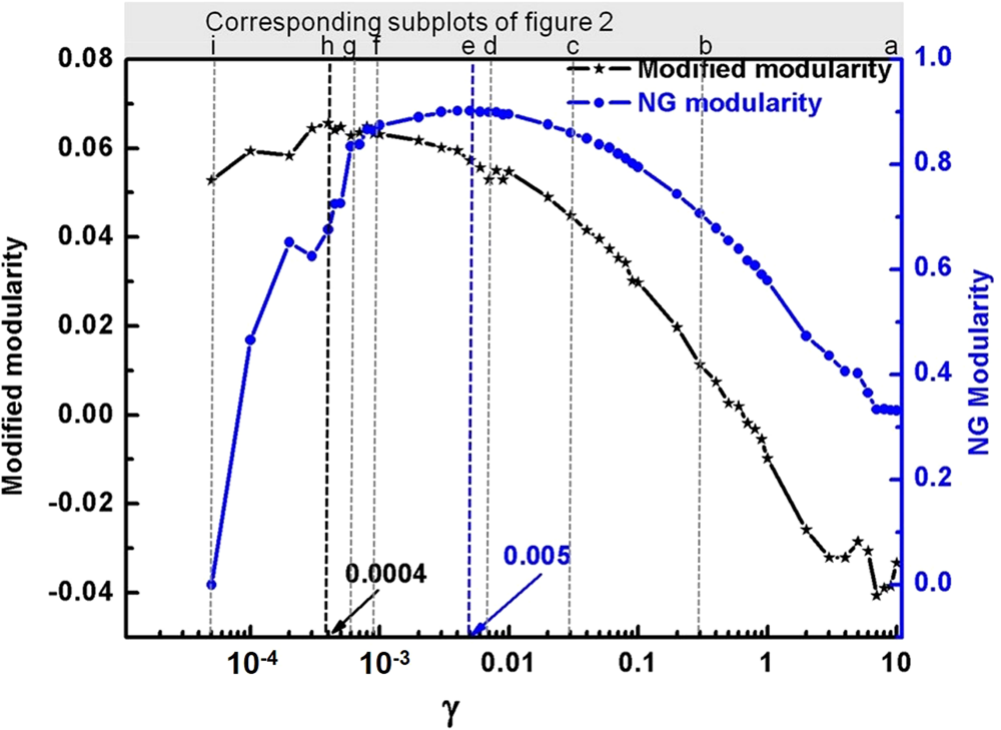
Modularity of community structures obtained at different *γ* values calculated using NG and our new modularity function of Eq. (3). Our function gives highest value at *γ* = 0.0004 (Fig. 2h, the right solution) whereas NG modularity has highest value at *γ* = 0.005 (Fig. 2e), clearly not the most optimal solution). The secondary x-axis represents indices of the subplots in Fig. 2.

The contact network (Fig. 1b) is created from the granular ensemble (Fig. 1a) as discussed in the ‘*DEM simulation to generate granular ensemble’* section of “Method”. The network is then partitioned at different *γ* values using RN model (Eq. (2)). We have depicted the communities obtained at different *γ* values (i.e at varying structural resolution), keeping the node position to be the same as particle coordinates (Fig. 2) to establish a visual correlation between the RN model output and the coordination number plot (Fig. 1a). As the *γ* reduces in size from 10 to 5 × 10^−5^ (*γ* is a dimensionless parameter), the average community size increases gradually. This is reflected in Fig. 2a–i. The γ parameter set the resolution scale. Minimizing Eq. (2) over range of γ values, systematically uncovers organized structures in the network at different length scale. It is vividly seen that Fig. 2h has highest visual correlation with the coordination number plot where the regular/ordered region are clearly separated from irregular/disordered nodes. Due to this clear demarcation, Fig. 2h is expected to have highest modularity among all the possible partitions. This aspect is investigated in Fig. 3.

It is important to note that the raw values of these two modularity functions (NG vs. our new modified modularity) should not be compared because any one of them can be shifted by an innocuous additive constant. It is the peak location that matters the most. It is evident from Fig. 3 that if community structure is separated at disordered regions then our method finds the most optimal partition (at *γ* = 0.0004, corresponding to Fig. 2h), whereas NG method failed to do so (the NG modularity suggested that the best partition occurs at *γ* = 0.005; corresponding to Fig. 2e).

### Results for PC 2

**Figure 4:**
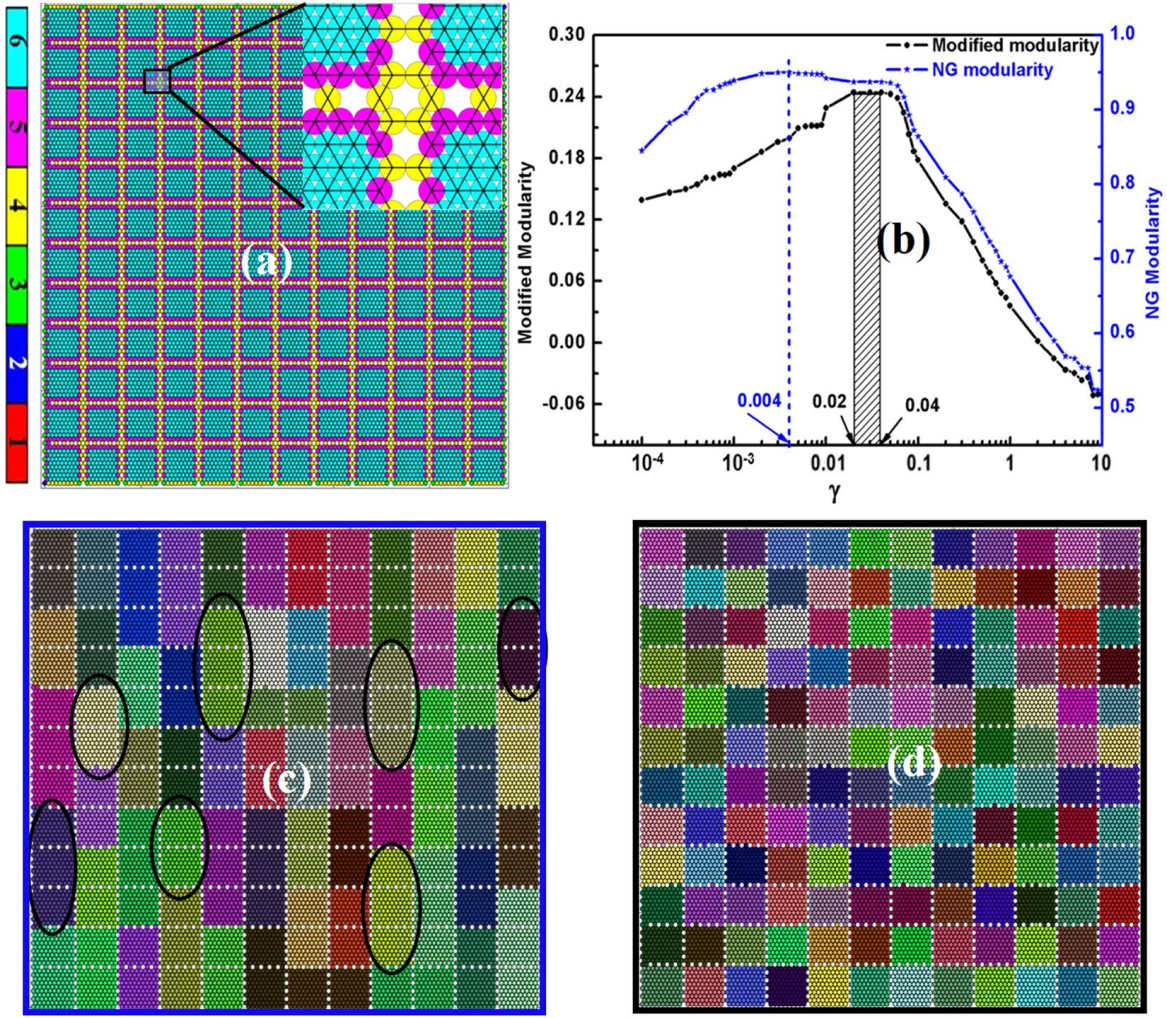
Comparison of best partition obtained using NG modularity vs. new modularity. **(a)** Synthetically generated granular ensemble with periodic distribution of disordered region. The particles are colored based on their coordination numbers (see the colorbar at the extreme left, which is essentially same as in Fig. 1a). (**b**) The NG and our new modularity of community structures obtained by RN model at different resolution scale. **(c)** The maximum modular community structure detected by NG method at *γ* = 0.004. It detected many higher-mode ordering as most optimal solution (few of them are encircled; which are clearly not the best solution) **(d)** Maximum modular community structure detected by our function. This structure is most stable in the range of 0.02 ≤ *γ* ≤ 0.04 due to its high modularity. Therefore it is clear that, even in its peak, NG modularity is picking mixed modes (circled regions in **c**), whereas the new function, in the range of 0.02 ≤ *γ* ≤ 0.04, is picking up only the pure primary mode.

We next analyzed a synthetically generated granular ensemble with periodic distribution of disordered regions (Fig. 4a; the inset highlighting the network at small scales). This approach creates clearly visible structures, with an in-built hierarchy. The communities were found via the RN model. As seen in Fig. 4b, the NG and our new modified modularity feature similar behavior for high γ values (small average community size). However, for large communities (at lower γ) they behave differently (Fig. 4b).

The best community structure obtained by NG and new modularity is depicted in Fig. 4c,d respectively. The new modularity shows very high visual correlation with natural structure and correctly picks the prime modes only (γ in the range of 0.02–0.04 in Fig. 4b, corresponding partition depicted in Fig. 4d), whereas the NG modularity is picking mixed modes (at γ = 0.004 Fig. 4b, partition shown in Fig. 4c).

### Results for PC 3

**Figure 5:**
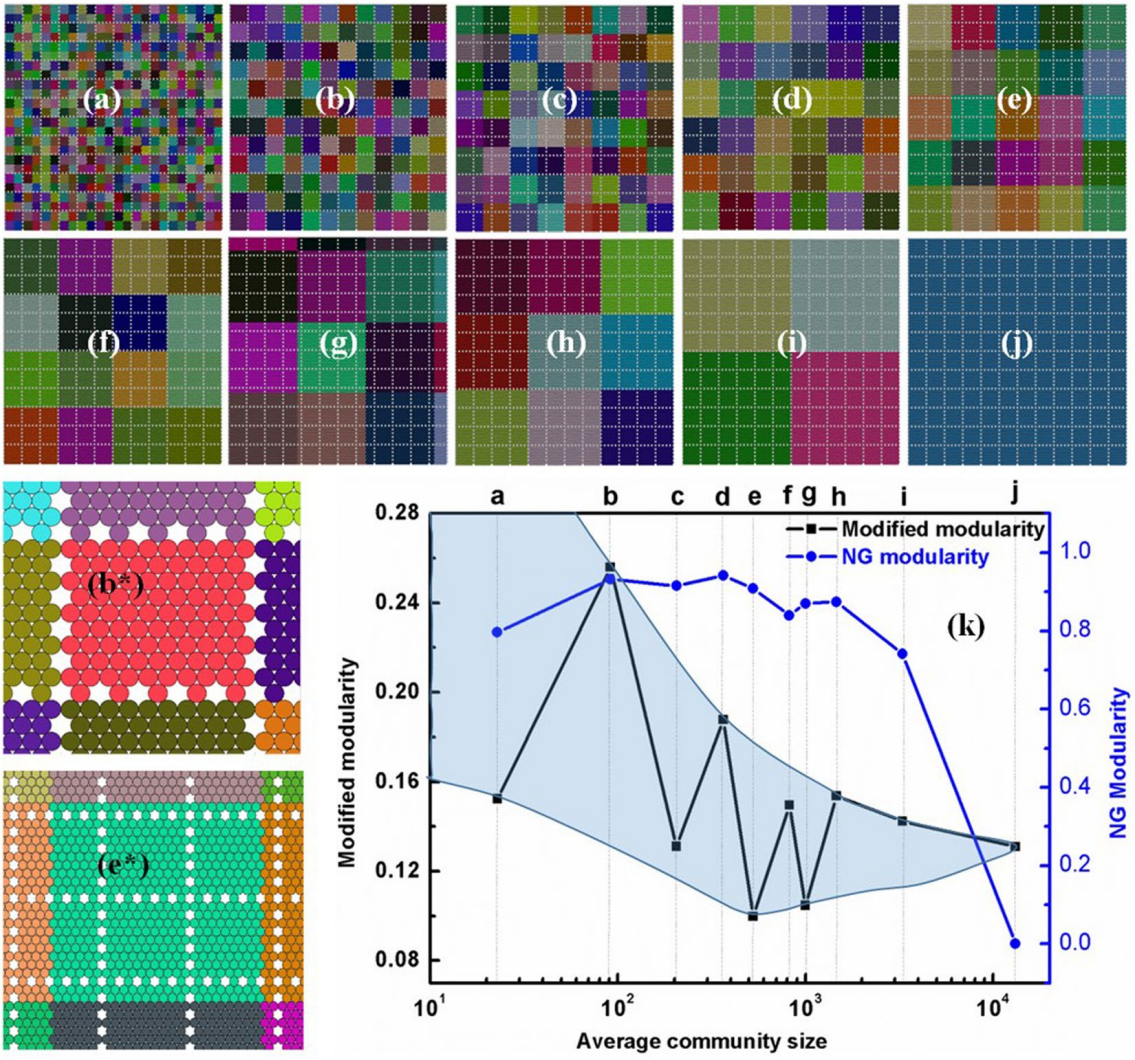
Manually partitioned network (**a–j)** and corresponding modularity obtained by NG and our new modularity function (as shown in (**k**)). Our function shows peaks for partition *b*, *d*, *f* and *h* and troughs for *a*, *c*, *e* and *g*; clearly indicating the presence of hierarchical community structures in the given network. Community separation for best (**b**) and worst (**e**) modular structures show that the corresponding partitions can be associated most and least with naturally identifiable structures respectively (b* and e* shows a magnified view of portions of *b* and *e* respectively, for clarity). The nature of these two functions is very different (clear from *k*) and demonstrates that, these two functions do not different by a simple constant.

Many real world networks contain hierarchical organization where strong groups of vertices have further sub-groups. Granular ensemble in Fig. 5a displays various sub-networks at different length scales. We have manually partitioned this network in roughly equal community sizes, which is so varied as to cover the entire length scale ranging from the primary mode length to system length (Fig. 5a to j, b* and e* shows a magnified view of best (b) and worst (e) modular structures). The peaks in our new modularity curve (Fig. 5k) confirm the presence of hierarchical structures in the given network. This illustrates that modular structures at different scales, can be identified by peaks in our modified modularity function. These correspond to the partitions where the community boundaries coincide with disordered regions and therefore help in identifying the higher modes clearly. The most modular structure (Fig. 5b) has all its communities separated through disordered regions (primary mode) whereas the lowest modular (Fig. 5e) has none of its communities separated through the disordered regions. Interestingly, the partition corresponding to Fig. 5f, exhibits a lower NG modularity than both Fig. 5e,g; by contrast, our new modified modularity function shows the opposite but correct trend. It is therefore clear that the NG- and our new- modified modularity functions are characteristically very different (not just by a constant). Our new modularity function is highly sensitive towards coincidence of the disordered regions with the community boundaries as reflected in the large variation in its modularity values (shaded region in modularity curve (Fig. 5k)) whereas NG modularity shows little variation in the range of prime importance. Therefore we establish that, our new modularity function, which is simple in structure and nature inspired, is very capable in finding the best partition, at least for the restricted classes of spherical granular media that are discussed in this article. Further studies are necessary to evaluate its performance in more generic spatial networks. Its high sensitivity to natural structural features can be exploited to discover inherent hierarchical structures, if present. If this method is proven to be useful in those generic spatial networks, then its simplicity and self-containment will make it a good choice for first-level unsupervised learning techniques targeted at spatially embedded networks.

## Conclusions

Here we have proposed a new modularity function that estimates the quality of partition of spatially embedded networks without employing a null model. Our new modularity function is inspired by the Hamiltonian of a spin-glass system. Notably, it incorporates geometric constraints through the use of a Heaviside function. Though arbitrary interactions may be incorporated, we employed couplings inspired from the null model (without explicitly using a null model). Our method accounts for the presence and absence (wherever applicable) of inter-community edges, which is neglected in nearly all kinds of formulations for modularity. The new modularity function is able to determine the “best” resolution scale at which the community structure has highest associability with the naturally identifiable structures. Our modularity function lucidly detected hierarchical structure present in the synthetically generated network with clearly its associated peaks and troughs as a function of the scale parameter; this was not the case for NG modularity. Taken together, our results suggest that the modified modularity function is more appropriate than the currently existing methods for unsupervised learning and analysis of spatially embedded networks.

## Supplementary information

Supplementary Information
