# Supplementary material for: A nature inspired modularity function for unsupervised learning involving spatially embedded networks
Source: Sci Rep. 2019 Feb 22;9:2631. doi: 10.1038/s41598-019-39180-8 (PMC6385190; doi:10.1038/s41598-019-39180-8)
Supplement: Supplementary file 1 — Supplementary Information [file 41598_2019_39180_MOESM1_ESM.pdf]

A nature inspired modularity function for unsupervised learning involving spatially embedded networks

Raj Kishore[1], Ajay K. Gogineni[2], Zohar Nussinov[3], Kisor K. Sahu[1*]

[1.] School of Minerals, Metallurgical and Materials Engineering, Indian Institute of Technology Bhubaneswar- 752050, India
[2.] School of Electrical Sciences, Indian Institute of Technology Bhubaneswar-752050, India
[3.] Department of Physics, Washington University in Saint Louis, MO- 63130-4899, USA

*Corresponding author: kisorsahu@iitbbs.ac.in

Supplementary Information (SI)

SI-1: Discrete Element Method (DEM) simulation details

Discrete Element Method (DEM) simulation is used to track the particle dynamics. It mainly models the collision between particles. The detailed information of packing algorithm which includes information of contact models and the numerical scheme used in the present DEM simulation can be found in [1]. Nevertheless, a brief summary is provided here for the sake of completeness and reproducibility of the article. All the important particle parameters used for the particle packing simulation are given in table S1.1.

Table S1.1: Particle Properties

Number of particles	N : 7428
Particle radius	R: 0.01 m
Young's modulus	Y: 10^9 Pa
Coefficient of friction	μ: 0.5
Damping constant	A : 0.01 sec
Tangential damping constant	γ^t : 10 Nsec/m
Material density	ρ_m: 8g/cm^3
Integration time step	Δt : 10^{-6} sec

Packing algorithm

In the present study soft particle DEM model is used where, particles motions are tracked by integration of the Newtonian equations of motion.

The translational and rotational motion of particle can be formulated as eqs. (S1.1) and (S1.2) respectively.

$$\frac{d\vec{v}}{dt} = \vec{F}/m \tag{S1.1}$$

$$\frac{d\vec{\omega}}{dt} = \vec{T}/I \tag{S1.2}$$

Where, $\vec{F}$ is the total force acting on the particle, *m* is the mass, $\vec{v}$ is velocity vector, $\vec{\omega}$ is the angular velocity vector, $\vec{T}$ is the net torque and *I* is the moment of inertia of the particle.

Modelling of contact forces

In soft particle DEM model, the non-zero overlap (ξ_{ij}, eq. S1.3) is given as

$$\xi_{ij} = R_i + R_j - |\vec{r}_i - \vec{r}_j| \tag{S1.3}$$

Where, R_i, R_j is the radius and $\vec{r}_i$, $\vec{r}_j$ is the position vector of the two interacting particles. The normal component of the contact force is modelled using Hertz model[2] which is further modified for viscoelastic spheres[3] and is given by the eq. S1.4.

$$F_{el}{}^{n} = \max\left\{0, \left[\frac{2\mathrm{Y}\sqrt{R^{eff}}}{3(1-\upsilon^2)}\left(\xi^{3/2} + A\sqrt{\xi}\frac{d\xi}{dt}\right)\right]\right\} \tag{S1.4}$$

The tangential force component has been modelled by using Haff and Werner model[4].

$$F^t = -sign(v_{rel}{}^t) \cdot \min\left(\gamma^t |v_{rel}{}^t|, \mu|F^n|\right) \tag{S1.5}$$

Where, γ^t is tangential damping constant and μ is the coefficient of friction.

Numerical scheme

Here we have used Gear integration scheme[5] for numerical integration. The flow chart of the DEM algorithm is given in figure S1.1. At the start of the simulation, all the required

parameters are initialised (time t =0). In the time increment loop (timestep of 1 μsec), the scheme performs three steps. **First** it predicts the values of all the parameters for the next time step by using Taylor series expansion. **Second** step is total force calculation on each particle. Total force on the particle is the sum of all the external forces and the interaction forces. Using the total force acting on each particle, one can calculate linear $\ddot{\vec{r}}^{corr}$ and angular accelerations by using Newton's law of motion (eq. S1.1 & eq. S1.2). **Third** step is the correction in the predicted values of first step. In this step, the difference in the two acceleration values, (eq. S4.6) is used to correct the predicted coordinates, predicted velocity and other higher order derivatives by using (eq. S1.7).

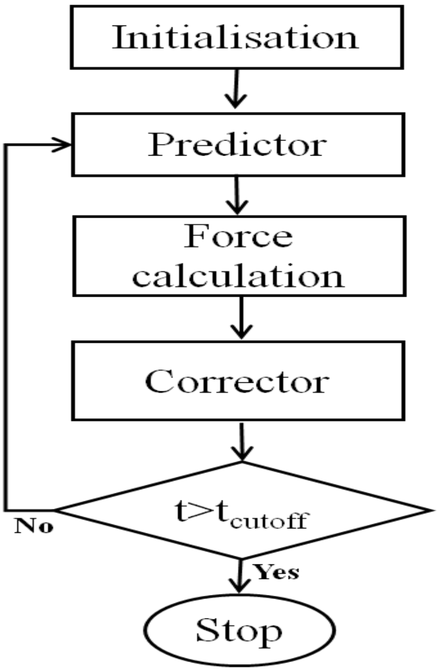

Fig. S1.1: DEM algorithm flow chart

$$\Delta\ddot{\vec{r}} = \ddot{\vec{r}}_i^{corr} - \ddot{\vec{r}}_i^{pr} \qquad \text{(S1.6)}$$

$$\begin{pmatrix} \vec{r}_i^{\,corr}(t+\Delta t) \\ \vec{V}_i^{\,corr}(t+\Delta t) \\ \ddot{\vec{r}}_i^{\,corr}(t+\Delta t) \\ \dddot{\vec{r}}_i^{\,corr}(t+\Delta t) \\ \bullet \\ \bullet \\ \bullet \\ \bullet \end{pmatrix} = \begin{pmatrix} \vec{r}_i^{\,pr}(t+\Delta t) \\ \vec{V}_i^{\,pr}(t+\Delta t) \\ \ddot{\vec{r}}_i^{\,pr}(t+\Delta t) \\ \dddot{\vec{r}}_i^{\,pr}(t+\Delta t) \\ \bullet \\ \bullet \\ \bullet \\ \bullet \end{pmatrix} + \begin{pmatrix} C_0 \\ C_1 \frac{1}{\Delta t} \\ C_2 \frac{2}{\Delta t^2} \\ C_3 \frac{6}{\Delta t^3} \\ \bullet \\ \bullet \\ \bullet \end{pmatrix} \bullet \frac{\Delta t^2}{2} \Delta \ddot{\vec{r}} \qquad \text{(S1.7)}$$

The coefficient c_i depends on the order of algorithm used and also on the type differential equation used. In the current simulation algorithm of fifth order is used for which the values of c_i are as given below-

C_0 = 19/90, C_1 =3/4, C_2=1, C_3 =1/2, C_4 =1/12 (S1.8)

The data is extracted and stored periodically at the interval of some predefined time steps. Simulation is terminated after a predefined time steps in all cases.

Boundary conditions

The static and dynamic properties of granular materials are greatly affected by the interaction of particle with the wall of the system. To eliminate this effect, the present simulation uses a hypothetical central force for particle movement. Here packing is obtained without using any container thus eliminating the use of any wall interaction with the particle. So the boundary conditions are not required for the present simulation.

SI-2: Algorithm (RN method)

RN method is based on spin-glass-type Potts model. It detects community by following two criterions: First, it favours the edges inside the community and second, it penalizes for the missing links inside the communities. Based on this consideration the Hamiltonian used in RN method is given as

$$H(\{\sigma\}) = -\frac{1}{2}\sum_{i \neq j}\left(a_{ij}A_{ij} - \gamma b_{ij}J_{ij}\right)\delta\left(\sigma_i, \sigma_j\right) \qquad \text{(S2.1)}$$

Here, the meaning of each term is same as used in eq. 2 of main article. The Hamiltonian of equation S2.1 is formulated based on the concept of formation of magnetic domains in magnetic material. It models a system, where all the connected spins of same magnetic domain interact ferromagnetically and if not connected then antiferromagnetically. The

equation S2.1 incorporates the contribution of these two interactions: “attractive” (ferromagnetic) and “repulsive” (anti-ferromagnetic) by using two different weight matrices: $a_{ij}A_{ij}$ and $b_{ij}J_{ij}$ respectively. The resolution parameter γ provides flexibility to adjust the energy exchange between these two interactions.

The algorithm of RN method considers each node separately. It iteratively decides the best community membership for a node by calculating the energy of the system and assigns the membership in which the system energy is lowest. A pictorial representation of RN algorithm is shown in figure S2.1. The steps of the algorithm[6] is discussed below-

(1) *System initialization:* The connection matrix A_{ij} and the edge weight a_{ij} and b_{ij} is initialised. This method starts with “symmetric“ state, where each node is assigned as separate community membership. So at the start of the simulation, the number of communities is same as the number of node.

(2) *Finding the best node membership:* Neighbor list of each node is generated. Calculate the energy change after moving the node into one of its connected cluster. Finally put this node in the cluster in which energy change is most negative (as it decreases the Hamiltonian)

(3) *Iterate until convergence*: Step (2) is repeated until there is no further new community membership assignment of any node is possible (minimum energy reached).

(4) *Test for the local energy minimum:*It might possible that the community structure obtained until step (3) is not the most modular structure. So in this step, we manually merge all the pair of connected communities. If this merging lowers the overall energy of the system consider this new merged community structure as best partition.

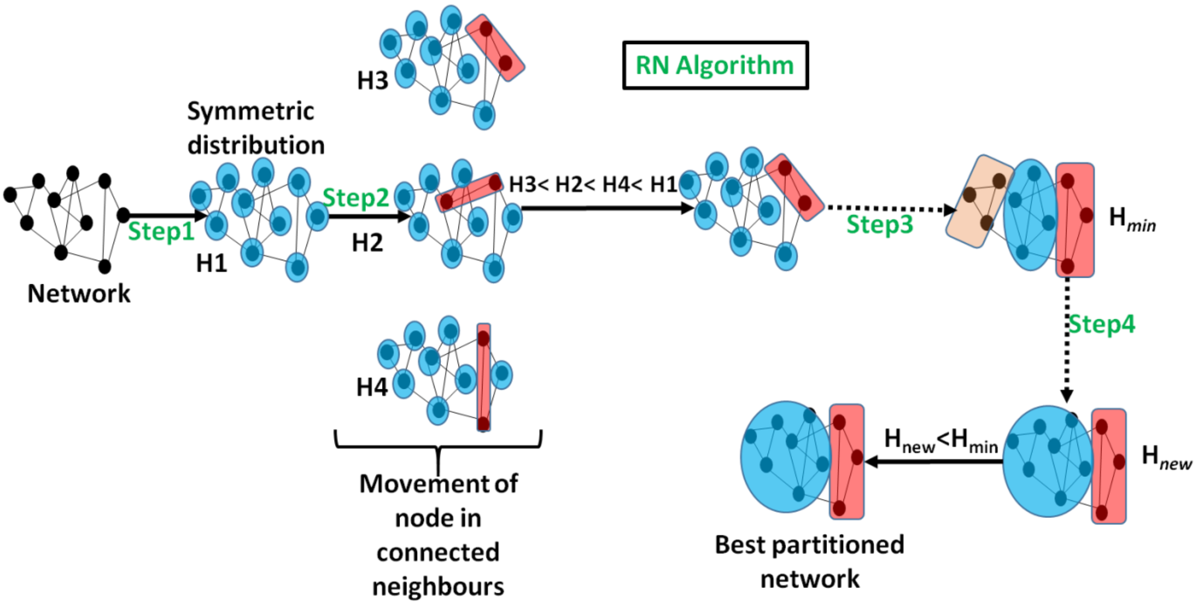

Fig S2.1: Pictorial representation of RN algorithm

SI-3: Different scenarios possible in network

In a partitioned network, there are four different scenarios are possible. A pictorial representation is shown in figure S3.1. These scenarios are based on the connected and missing edges, within or outside of the community. These are discussed below with their effect on the overall energy of the system:

1. An edge between nodes of same community. This decreases the energy of the system as it strenthen the community.
2. A missing edge inside the community. This increases the energy of the system as ideally the entites of same community should interact more frequently.
3. A missing edge between two different communities. This decreases the energy because entities of two different communities should not interact with each other. This will preserve the identity of each individual communities.
4. An edge between two different communities. This increases the energy of the system as this edge will reduce the clarity of boundaries between the communities.

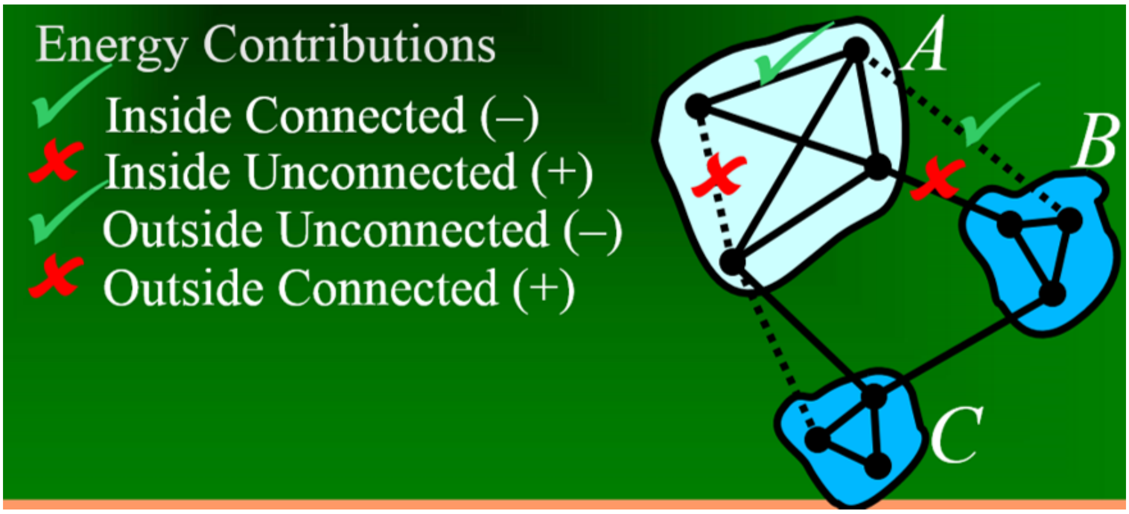

Fig. S3.1: Four scenarios possible in a partitioned network and its effect on the overall energy of the network. A, B and C are the three clusters of given network and tick (√) denotes the edges which reduces the energy and cross (x) indicates the edges which are increasing the energy of the system.

SI-4: Response of new modularity function for all possible scenarios

The modified modularity function is given as

$$Q(\sigma)=\frac{1}{2m}\sum_{i,j}\left(a_{ij}A_{ij}-\theta(\Delta x_{ij})\left|b_{ij}\right|J_{ij}\right)\left(2\delta(\sigma_i,\sigma_j)-1\right) \qquad \text{(S4.1)}$$

Here, the meaning of each term is same as used in eq. 3 of main article. It has four independent variables namely, a_{ij}, A_{ij}, $\theta(\Delta x_{ij})$ and $\delta(\sigma_i,\sigma_j)$. Each variable has two different possible values, so there will be 2^4=16 possible combinations of their values. In this section we have discussed the effect of these different combinations on $Q(\sigma)$ *i.e* whether it increase or decrease $Q(\sigma)$. A sample granular assembly has taken for pictorial representation of different cases if possible. Particles with different coordination numbers are differentiated by colors as reflected by color bar used in main article. The granular network obtained from the given ensemble will have same degree distribution as its coordination number distribution. The average degree for this network is 5.6. Maximum and minimum degree is 6 and 2 respectively. The cutoff distance is 2.1 times the radius of particles. Since this cutoff distance is nearly equal to the first neighbourhood of the particles, all those cases that considered an edge between nodes outside the cut-off distance (case 2, 4, 6, and 8) are physically not possible in the considered network. Case 9 and 11 is not possible because it considers two highly linked nodes (a_{ij}>0) within cut-off distance to be unconnected.

Case 1: Node *i* and *j* connected, within cut-off range and in same community

$a_{ij}>0$, $A_{ij}=1$, $\delta(\sigma_i,\sigma_j)=1$, $\theta(\Delta x_{ij})=1$, $J_{ij}=0$, $b_{ij}>0$

$a_{ij}=6-5.6>0$, $\theta(\Delta x_{ij})=1$, since Δx_{ij} =2.1r-1.97r>0 (r is radius of particle)

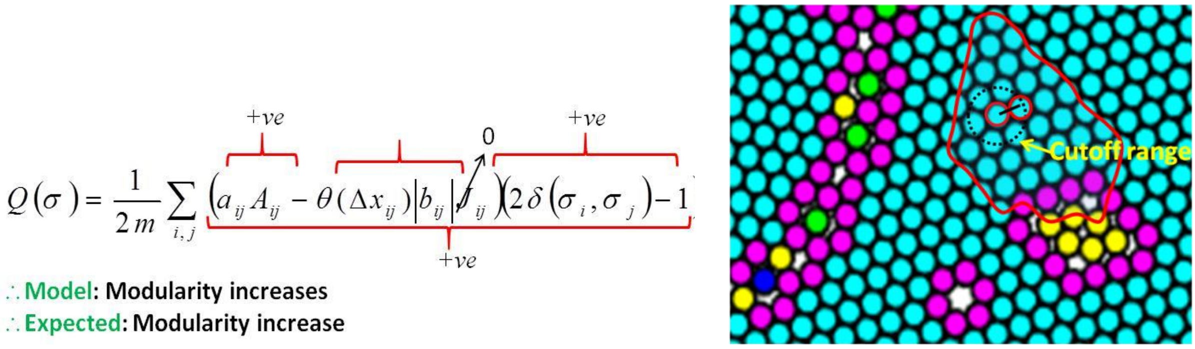

This case is similar to the first scenario that we have discussed in S3. As this edge is between two highly linked nodes (a_{ij}>0) will strengthen the community, it should decrease the overall modularity of the system. Thus our function output is matching with the expected output.

Case 2 (not possible in considered network): Node *i* and *j* connected, outside cut-off range and in same community

$a_{ij}>0$, $A_{ij}=1$, $\delta(\sigma_i,\sigma_j)=1$, $\theta(\Delta x_{ij})=0$, $J_{ij}=0$, $b_{ij}>0$

a_{ij}=6-5.6>0, θ(Δx_{ij})=0, since Δx_{ij} =2.1r-4r<0 (r is radius of particle)

$$Q(\sigma) = \frac{1}{m}\sum_{i,j}\left(a_{ij}A_{ij} - \theta(\Delta x_{ij})|b_{ij}|J_{ij}\right)\left(2\delta(\sigma_i,\sigma_j)-1\right)$$

+ve, 0, 0, +ve; +ve

∴ **Model**: Modularity increases

∴ **Expected**: Modularity increase

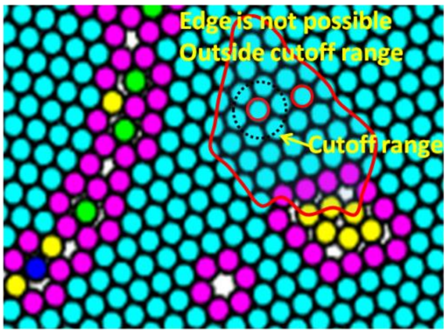

The output of our function for this case favors an edge within the same community. Our function value is positive because a_{ij} is positive, which indicates that the two connected nodes are high degree nodes. So putting highly linked nodes in same community should increase the modularity.

Case 3: Node *i* and *j* connected, within cut-off range and in different community

a_{ij}>0, A_{ij}=1, δ(σ_i,σ_j)=0, θ(Δx_{ij})=1, J_{ij}=0, b_{ij}>0

a_{ij}=6-5.6>0, θ(Δx_{ij})=1, since Δx_{ij} =2.1r-1.97r>0 (r is radius of particle)

$$Q(\sigma) = \frac{1}{2m}\sum_{i,j}\left(a_{ij}A_{ij} - \theta(\Delta x_{ij})|b_{ij}|J_{ij}\right)\left(2\delta(\sigma_i,\sigma_j)-1\right)$$

+ve, 0, -ve; -ve

∴ **Model**: Modularity decreases

∴ **Expected**: Modularity decrease

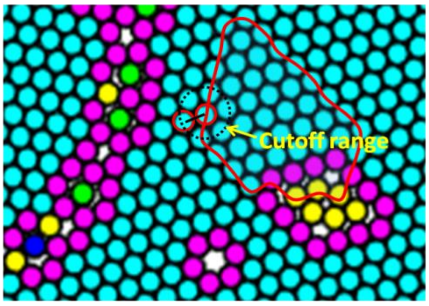

This case is similar to the fourth scenario discussed in S3. Links between two different communities should always be restricted because the connected nodes are highly linked nodes (a_{ij}>0). Thus this arrangement should decrease the modularity of the system. Our function is giving the same output as expected.

Case 4 (not possible in considered network): Node *i* and *j* connected, outside cut-off range and in different community

a_{ij}>0, A_{ij}=1, δ(σ_i,σ_j)=0, θ(Δx_{ij})=0, J_{ij}=0, b_{ij}>0

a_{ij}=6-5.6>0, θ(Δx_{ij})=0, since Δx_{ij} =2.1r-3.7r<0 (r is radius of particle)

$$Q(\sigma) = \frac{1}{2m}\sum_{i,j}\left(a_{ij}A_{ij} - \theta(\Delta x_{ij})|b_{ij}|J_{ij}\right)\left(2\delta(\sigma_i,\sigma_j)-1\right)$$

+ve, 0, 0, -ve; -ve

∴ **Model**: Modularity decreases

∴ **Expected**: Modularity decrease

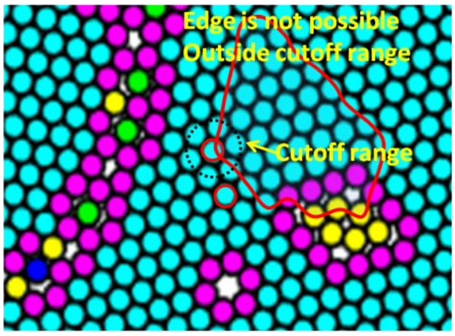

In this case also there is an inter-community link which is in between two highly linked nodes (a_{ij}>0), which should not be favoured. Thus this arrangement should decrease the modularity of the system and our function is doing the same.

Case 5: Node *i* and *j* connected, within cut-off range and in same community

$a_{ij}<0$, $A_{ij}=1$, $\delta(\sigma_i,\sigma_j)=1$, $\theta(\Delta x_{ij})=1$, $J_{ij}=0$, $b_{ij}>0$

$a_{ij}=5-5.6<0$, $\theta(\Delta x_{ij})=1$, since Δx_{ij} =2.1r-1.97r>0 (r is radius of particle)

$$Q(\sigma)=\frac{1}{2m}\sum_{i,j}\underbrace{\left(\overbrace{a_{ij}A_{ij}}^{-ve}-\overbrace{\theta(\Delta x_{ij})\left|b_{ij}\right|\overset{0}{J_{ij}}}\right)\overbrace{\left(2\delta(\sigma_i,\sigma_j)-1\right)}^{+ve}}_{-ve}$$

∴ Model: Modularity decreases
∴ Expected: Modularity decrease

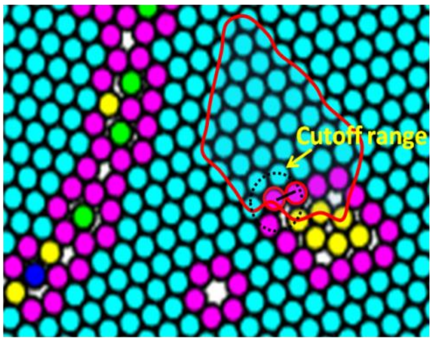

In this case though edge is in between nodes of same community, it should not be favored as these two nodes are low degree nodes (boundary nodes, a_{ij}<0) and should ideally be treated as a separate community that can be thought as "boundary community". The strength of our method is its capability to distinguish such "boundary communities".

Case 6 (not possible in considered network): Node *i* and *j* connected, outside cut-off range and in same community

$a_{ij}<0$, $A_{ij}=1$, $\delta(\sigma_i,\sigma_j)=1$, $\theta(\Delta x_{ij})=0$, $J_{ij}=0$, $b_{ij}>0$

$a_{ij}=5-5.6<0$, $\theta(\Delta x_{ij})=0$, since Δx_{ij} =2.1r-4r<0 (r is radius of particle)

$$Q(\sigma)=\frac{1}{2m}\sum_{i,j}\underbrace{\left(\overbrace{a_{ij}A_{ij}}^{-ve}-\overbrace{\overset{0}{\theta}(\Delta x_{ij})\left|b_{ij}\right|\overset{0}{J_{ij}}}\right)\overbrace{\left(2\delta(\sigma_i,\sigma_j)-1\right)}^{+ve}}_{-ve}$$

∴ Model: Modularity decreases
∴ Expected: Modularity decrease

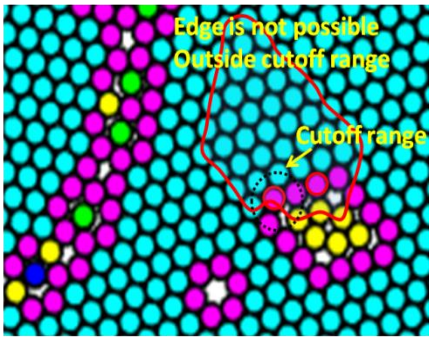

This case is similar to case 1 which is discussed above, only the difference is that the connected nodes are low degree nodes (a_{ij}<0). Thus putting them in same community might decrease the strength of the community. So the community membership of these two connected nodes should be different.

Case 7: Node *i* and *j* connected, within cut-off range and in different community

$a_{ij}<0$, $A_{ij}=1$, $\delta(\sigma_i,\sigma_j)=0$, $\theta(\Delta x_{ij})=1$, $J_{ij}=0$, $b_{ij}>0$

$a_{ij}=4-5.6<0$, $\theta(\Delta x_{ij})=1$, since Δx_{ij} =2.1r-1.97r>0 (r is radius of particle)

$$Q(\sigma)=\frac{1}{2m}\sum_{i,j}\left(a_{ij}A_{ij}-\theta(\Delta x_{ij})\left|b_{ij}\right|J_{ij}\right)\left(2\delta(\sigma_i,\sigma_j)-1\right)$$

-ve, 0, -ve, +ve

∴ Model: Modularity increases
∴ Expected: Modularity increase

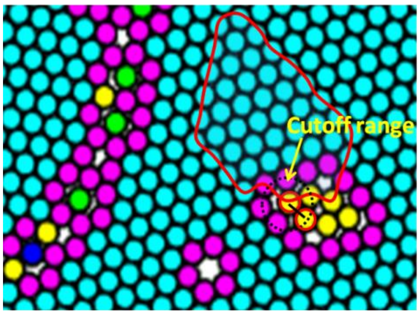

In this case, two connected lower degree (($a_{ij}<0$) nodes are having different community membership. This should increase the modularity and our function is giving the same output as expected.

Case 8 (not possible in considered network): Node *i* and *j* connected, outside cut-off range and in different community

$a_{ij}<0$, $A_{ij}=1$, $\delta(\sigma_i,\sigma_j)=0$, $\theta(\Delta x_{ij})=0$, $J_{ij}=0$, $b_{ij}>0$

$a_{ij}=4.5-5.6<0$, $\theta(\Delta x_{ij})=0$, since Δx_{ij} =2.1r-4.2r<0 (r is radius of particle)

$$Q(\sigma)=\frac{1}{2m}\sum_{i,j}\left(a_{ij}A_{ij}-\theta(\Delta x_{ij})\left|b_{ij}\right|J_{ij}\right)\left(2\delta(\sigma_i,\sigma_j)-1\right)$$

-ve, 0, 0, -ve, +ve

∴ Model: Modularity increases
∴ Expected: Modularity increase

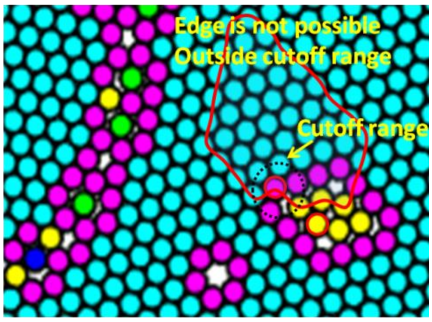

This case is similar to the previous case, where two connected lower degree (($a_{ij}<0$) nodes are having different community membership. This should increase the modularity and our function is giving the same output as expected.

Case 9 (not possible in considered network): Node *i* and *j* not connected, within cut-off range and in same community

$a_{ij}>0$, $A_{ij}=0$, $\delta(\sigma_i,\sigma_j)=1$, $\theta(\Delta x_{ij})=1$, $J_{ij}=1$, $b_{ij}>0$

$a_{ij}=6-5.6>0$, $\theta(\Delta x_{ij})=1$, since Δx_{ij} =2.1r-1.97r>0 (r is radius of particle)

$$Q(\sigma)=\frac{1}{2m}\sum_{i,j}\underbrace{\left(a_{ij}A_{ij}-\theta(\Delta x_{ij})|b_{ij}|J_{ij}\right)}_{-ve}\overbrace{\left(2\delta(\sigma_i,\sigma_j)-1\right)}^{+ve}$$

∴ Model: Modularity decreases

∴ Expected: Modularity decrease

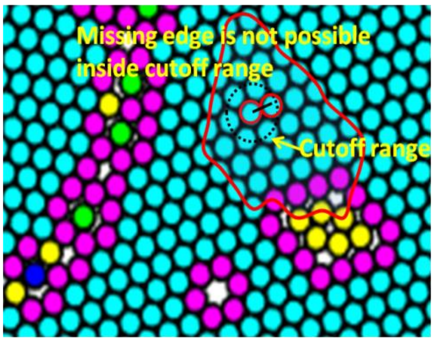

In this case two highly linked nodes (a_{ij}>0) in the same community are unconnected.This should not be favoured as it weaken the community strength. This is incorporated in our function as it is reducing the modularity value.

Case 10: Node *i* and *j* not connected, outside cut-off range and in same community

$a_{ij}>0$, $A_{ij}=0$, $\delta(\sigma_i,\sigma_j)=1$, $\theta(\Delta x_{ij})=0$, $J_{ij}=1$, $b_{ij}>0$

$a_{ij}=6-5.6>0$, $\theta(\Delta x_{ij})=0$, since Δx_{ij} =2.1r-4r<0 (r is radius of particle)

$$Q(\sigma)=\frac{1}{2m}\sum_{i,j}\underbrace{\left(a_{ij}A_{ij}-\theta(\Delta x_{ij})|b_{ij}|J_{ij}\right)}_{0}\overbrace{\left(2\delta(\sigma_i,\sigma_j)-1\right)}^{+ve}$$

∴ Model: No change in modularity

∴ Expected: No change in modularity

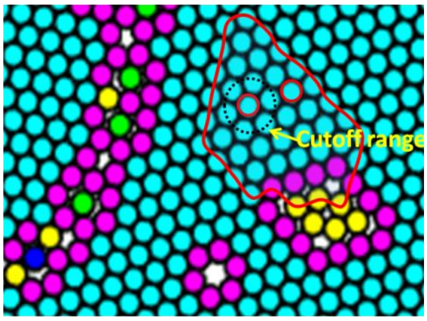

The output of our function depict that it is restricting the over penalization of those missing links which are physically neither possible nor to be expected. Our function is showing no change for the existence of these missing links inside the community.

Case 11 (not possible in considered network): Node *i* and *j* not connected, within cut-off range and in different community

$a_{ij}>0$, $A_{ij}=0$, $\delta(\sigma_i,\sigma_j)=0$, $\theta(\Delta x_{ij})=1$, $J_{ij}=1$, $b_{ij}>0$

$a_{ij}=6-5.6>0$, $\theta(\Delta x_{ij})=1$, since Δx_{ij} =2.1r-1.97r>0 (r is radius of particle)

$$Q(\sigma)=\frac{1}{2m}\sum_{i,j}\underbrace{\left(a_{ij}A_{ij}-\theta(\Delta x_{ij})|b_{ij}|J_{ij}\right)}_{+ve}\overbrace{\left(2\delta(\sigma_i,\sigma_j)-1\right)}^{-ve}$$

∴ Model: Modularity increases

∴ Expected: Modularity increase

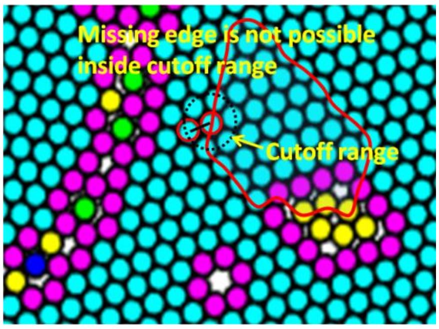

In this case existence of a missing link between two different communities is favoured. It should increase the modularity of the system and our function output is matches with the expected output.

Case 12: Node *i* and *j* not connected, outside cut-off range and in different community

$a_{ij}>0$, $A_{ij}=0$, $\delta(\sigma_i,\sigma_j)=0$, $\theta(\Delta x_{ij})=0$, $J_{ij}=1$, $b_{ij}>0$

$a_{ij}=6-5.6>0$, $\theta(\Delta x_{ij})=0$, since Δx_{ij} =2.1r-3.7r<0 (r is radius of particle)

$$Q(\sigma)=\frac{1}{2m}\sum_{i,j}\underbrace{\left(\overbrace{a_{ij}A_{ij}}-\overbrace{\theta(\Delta x_{ij})\left|b_{ij}\right|J_{ij}}\right)\overbrace{\left(2\delta(\sigma_i,\sigma_j)-1\right)}^{-ve}}_{0}$$

∴ Model: No change in modularity

∴ Expected: No change in modularity

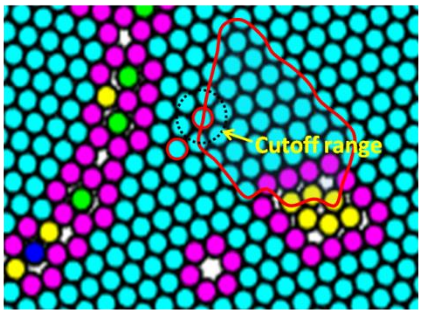

In this case our function is showing no change in the modularity as the two considered nodes are outside the cutoff distance and not connected, which is a realistic condition. So there should not be any change in the modularity.

Case 13: Node *i* and *j* not connected, within cut-off range and in same community

$a_{ij}<0$, $A_{ij}=0$, $\delta(\sigma_i,\sigma_j)=1$, $\theta(\Delta x_{ij})=1$, $J_{ij}=1$, $b_{ij}>0$

$a_{ij}=4.5-5.6<0$, $\theta(\Delta x_{ij})=1$, since Δx_{ij} =2.1r-2.05r>0 (r is radius of particle)

$$Q(\sigma)=\frac{1}{2m}\sum_{i,j}\underbrace{\left(\overbrace{a_{ij}A_{ij}}-\overbrace{\theta(\Delta x_{ij})\left|b_{ij}\right|J_{ij}}\right)\overbrace{\left(2\delta(\sigma_i,\sigma_j)-1\right)}^{+ve}}_{-ve}$$

∴ Model: Modularity decreases

∴ Expected: Modularity decrease

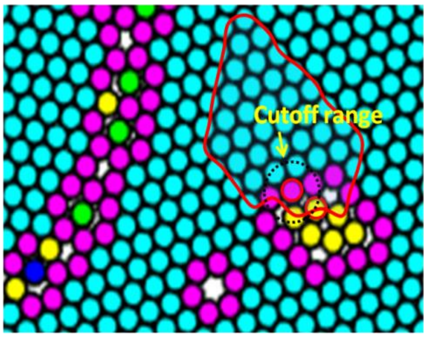

In this case two nodes are unconnected inside the same community. This should reduce the modularity of the system. Our function is also decreasing the modularity value.

Case 14: Node *i* and *j* not connected, outside cut-off range and in same community

$a_{ij}<0$, $A_{ij}=0$, $\delta(\sigma_i,\sigma_j)=1$, $\theta(\Delta x_{ij})=0$, $J_{ij}=1$, $b_{ij}>0$

$a_{ij}=4.5-5.6<0$, $\theta(\Delta x_{ij})=0$, since Δx_{ij} =2.1r-3r<0 (r is radius of particle)

$$Q(\sigma)=\frac{1}{2m}\sum_{i,j}\underbrace{\left(a_{ij}A_{ij}-\theta(\Delta x_{ij})\left|b_{ij}\right|J_{ij}\right)\overbrace{\left(2\delta(\sigma_i,\sigma_j)-1\right)}^{+ve}}_{0}$$

∴ Model: No change in modularity
∴ Expected: No change in modularity

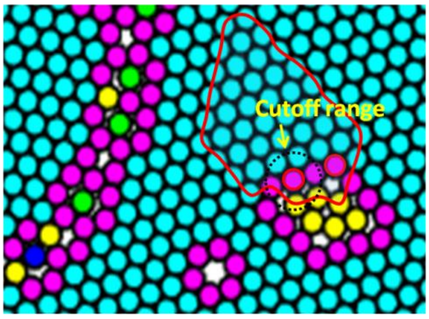

In this case two nodes are outside the cutoff distance and not connected, which is a realistic condition. So there should not be any change in the modularity.

Case 15: Node *i* and *j* not connected, within cut-off range and in different community

$a_{ij}<0$, $A_{ij}=0$, $\delta(\sigma_i,\sigma_j)=0$, $\theta(\Delta x_{ij})=1$, $J_{ij}=1$, $b_{ij}>0$

$a_{ij}=4.5-5.6<0$, $\theta(\Delta x_{ij})=1$, since $\Delta x_{ij}=2.1r-2.05r>0$ (r is radius of particle)

$$Q(\sigma)=\frac{1}{2m}\sum_{i,j}\underbrace{\left(a_{ij}A_{ij}-\theta(\Delta x_{ij})\left|b_{ij}\right|J_{ij}\right)\overbrace{\left(2\delta(\sigma_i,\sigma_j)-1\right)}^{-ve}}_{+ve}$$

∴ Model: Modularity increases
∴ Expected: Modularity increase

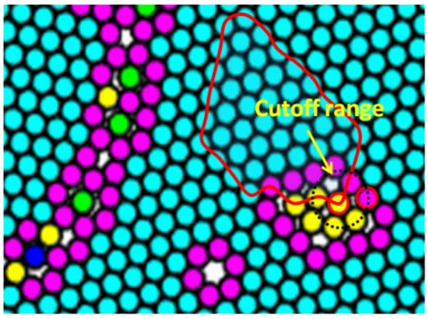

In this case two considered nodes are not connected and within cut-off distance and also in the same community. This should increase the modularity value and our function is doing the same thing.

Case 16: Node *i* and *j* not connected, outside cut-off range and in different community

$a_{ij}<0$, $A_{ij}=0$, $\delta(\sigma_i,\sigma_j)=0$, $\theta(\Delta x_{ij})=0$, $J_{ij}=1$, $b_{ij}>0$

$a_{ij}=4.5-5.6<0$, $\theta(\Delta x_{ij})=0$, since $\Delta x_{ij}=2.1r-4r<0$ (r is radius of particle)

$$Q(\sigma)=\frac{1}{2m}\sum_{i,j}\underbrace{\left(a_{ij}A_{ij}-\theta(\Delta x_{ij})\left|b_{ij}\right|J_{ij}\right)\overbrace{\left(2\delta(\sigma_i,\sigma_j)-1\right)}^{-ve}}_{0}$$

∴ Model: No change in modularity
∴ Expected: No change in modularity

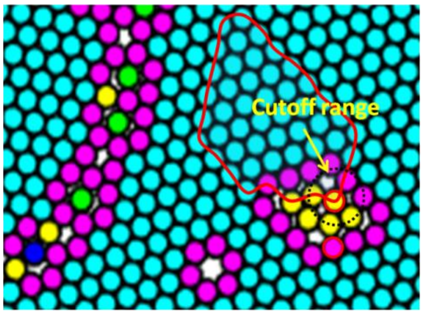

In this case two nodes outside the cutoff distance should not be connected. They are also in different community. So it should not alter the modularity value.

Table S4.1: Summary

Parameters	a_{ij}	A_{ij}	$\delta(\sigma_i, \sigma_j)$	θ	Q(model)	Q(expected)
Case -1	+	1	1	1	+	+
Case -2	+	1	1	0	+	+
Case -3	+	1	0	1	-	-
Case -4	+	1	0	0	-	-
Case -5	-	1	1	1	-	-
Case -6	-	1	1	0	-	-
Case -7	-	1	0	1	+	+
Case -8	-	1	0	0	+	+
Case -9	+	0	1	1	-	-
Case -10	+	0	1	0	0	0
Case -11	+	0	0	1	+	+
Case -12	+	0	0	0	0	0
Case -13	-	0	1	1	-	-
Case -14	-	0	1	0	0	0
Case -15	-	0	0	1	+	+
Case -16	-	0	0	0	0	0

References

[1] Kishore, R. Das, S. Nussinov, Z. and Sahu K. K. Kinetic instability, symmetry breaking and role of geometric constraints on the upper bounds of disorder in two dimensional packings. *Sci. Rep.* **6**, srep26968. (2016).

[2] Hertz, H. Über die Berührung fester elastischer Körper. Journal für die reine und angewandte Mathematik, **92**, 156-171 (1882).

[3] Brilliantov, N., Spahn, F., Hertzsch, J. & Pöschel, T. Model for collisions in granular gases. *Phy. Rev. E* **53**, 5382-5392 (1996).

[4] Haff, P. & Werner, B. Computer simulation of the mechanical sorting of grains. *Powder Technol*. **48**, 239-245 (1986)

[5] Gear, C. W. The numerical integration of ordinary differential equations. *Math. Comput.* **21**, 146–156 (1967).

[6] Ronhovde, Peter. Physical models in community detection with applications to identifying structure in complex amorphous systems. ProQuest Dissertations Publishing (2010).
